# Metabolome Alterations Linking Sugar-Sweetened Beverage Intake with Dyslipidemia in Youth: The Exploring Perinatal Outcomes among CHildren (EPOCH) Study

**DOI:** 10.3390/metabo12060559

**Published:** 2022-06-17

**Authors:** Catherine C. Cohen, Dana Dabelea, Gregory Michelotti, Lu Tang, Kartik Shankar, Michael I. Goran, Wei Perng

**Affiliations:** 1 Department of Pediatrics, School of Medicine, University of Colorado Anschutz Medical Campus, Aurora, CO 80045, USA; dana.dabelea@cuanschutz.edu (D.D.); kartik.shankar@cuanschutz.edu (K.S.); 2 Lifecourse Epidemiology of Adiposity and Diabetes (LEAD) Center, University of Colorado Denver Anschutz Medical Campus, Aurora, CO 80045, USA; wei.perng@cuanschutz.edu; 3 Department of Epidemiology, Colorado School of Public Health, University of Colorado Anschutz Medical Campus, Aurora, CO 80045, USA; 4 Metabolon, Inc., Morrisville, NC 27560, USA; gmichelotti@metabolon.com; 5 Department of Biostatistics, School of Public Health, University of Pittsburgh, Pittsburgh, PA 15261, USA; lutang@pitt.edu; 6 Department of Pediatrics, Children’s Hospital Los Angeles, Keck School of Medicine, University of Southern California, Los Angeles, CA 90007, USA; goran@usc.edu; 7 Department of Nutritional Sciences, University of Michigan School of Public Health, Ann Arbor, MI 48109, USA

**Keywords:** added sugar, metabolic profiling, childhood obesity, machine learning, metabolic syndrome, hypertriglyceridemia

## Abstract

The objective of this study was to assess intermediary metabolic alterations that link sugar-sweetened beverage (SSB) intake to cardiometabolic (CM) risk factors in youth. A total of 597 participants from the multi-ethnic, longitudinal Exploring Perinatal Outcomes among CHildren (EPOCH) Study were followed in childhood (median 10 yrs) and adolescence (median 16 yrs). We used a multi-step approach: first, mixed models were used to examine the associations of SSB intake in childhood with CM measures across childhood and adolescence, which revealed a positive association between SSB intake and fasting triglycerides (β (95% CI) for the highest vs. lowest SSB quartile: 8.1 (−0.9,17.0); *p*-trend = 0.057). Second, least absolute shrinkage and selection operator (LASSO) regression was used to select 180 metabolite features (out of 767 features assessed by untargeted metabolomics) that were associated with SSB intake in childhood. Finally, 13 of these SSB-associated metabolites (from step two) were also prospectively associated with triglycerides across follow-up (from step one) in the same direction as with SSB intake (Bonferroni-adj. *p* < 0.0003). All annotated compounds were lipids, particularly dicarboxylated fatty acids, mono- and diacylglycerols, and phospholipids. In this diverse cohort, we identified a panel of lipid metabolites that may serve as intermediary biomarkers, linking SSB intake to dyslipidemia risk in youth.

## 1. Introduction

Excess consumption of sugar-sweetened beverages (SSBs), which typically include sodas, fruit-flavored drinks, energy drinks, sports drinks, and sweetened teas/coffees, has been consistently associated with rates of overweight and obesity among youth over recent decades [1,2,3]. This associated weight gain may result from the energy-dense, liquid nature of SSBs, resulting in weaker satiation and compensatory food responses, or activation of the hedonic food reward system [4]. SSB intake has also been associated with cardiometabolic risk factors in childhood and adolescence, including markers of insulin resistance, inflammation, and dyslipidemia, independent of energy intake [5,6,7,8,9,10]. Proposed mechanisms include the higher glycemic index of SSBs, which may contribute to an increased insulin secretory response [11], or associations of added sugars in SSBs, especially fructose, with hepatic de novo lipogenesis and ectopic liver fat [12,13,14,15,16], an effect that has been observed even when weight is held stable [13]. A critical future direction is, therefore, better understanding the pathophysiological disturbances associated with SSBs, as this may also shed light on objective biomarkers of intake of SSBs, a food group for which social norms may bias self-reported intake data.

Metabolomics is an evolving science that involves the comprehensive measurement of low molecular weight molecules, or metabolites, in biological samples. This includes endogenous compounds, which serve as products, intermediates, and substrates of chemical reactions in the human metabolism, as well as exogenous compounds, which reflect environmental exposures [17]. In adults, a study by Gibbons et al. leveraged untargeted metabolomics to identify a set of metabolites (formate, citrulline, taurine, isocitrate) that showed promise as biomarkers of SSB intake [18], though this study did not link these metabolites to any health outcomes. An alternative strategy is to evaluate the metabolome alterations linking SSB intake and cardiometabolic risk. This may be achieved by using a “meet-in-the-middle” approach [19], an analytical framework that aims to identify the functional biomarkers that mark the relationship between an exposure and a health outcome, thereby providing insight into pathophysiological alterations potentially attributable to the exposure. For example, in a cross-sectional cohort of Mexican youth, Perng et al. identified two metabolites (urate and nonanoate) that marked the relationship between SSB intake and higher blood pressure in girls [20]. While these findings serve as a foundation for understanding the link between SSB intake and one metabolic risk factor in youth, additional prospective studies are needed to further explore this pathway and establish the temporality of associations.

**Figure 1:**
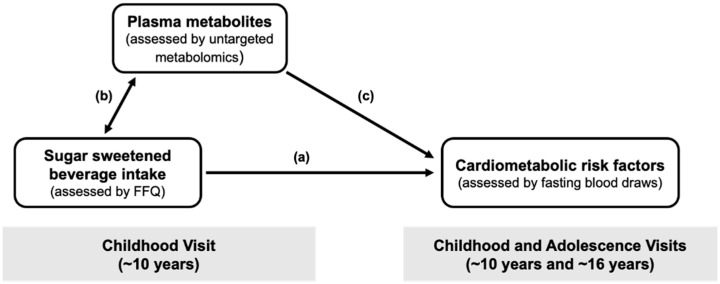
Conceptual diagram of the “meet in the middle” approach used to identify metabolites that mark the relationship between sugar sweetened beverage (SSB) intake and cardiometabolic risk. (**a**) In step 1, we tested prospective associations of SSB intake in childhood with cardiometabolic risk factors across childhood and adolescence. (**b**) In step 2, we identified plasma metabolites in childhood associated with SSB intake in childhood. (**c**) In step 3, we tested whether SSB-associated metabolites (from step 2) were also prospectively associated with SSB-associated CM risk factors across childhood and adolescence (from step 1). Abbreviations: FFQ, food frequency questionnaire; SSB, sugar-sweetened beverage.

In this study, we investigated the intermediary metabolic alterations that mark the relationship between SSB intake in childhood and cardiometabolic risk factors measured prospectively across childhood and adolescence. This was done using data from the Exploring Perinatal Outcomes among CHildren (EPOCH) study, a longitudinal cohort of diverse youth, and by employing a multi-step conceptual framework, summarized in Figure 1, which integrated data collected during childhood and/or adolescence on diet, metabolomics, and conventional cardiometabolic measures [fasting glucose and insulin, insulin resistance (assessed using the homeostatic model of assessment), HDL cholesterol, fasting triglycerides, and systolic blood pressure].

## 2. Results

### 2.1. Characteristics

Background characteristics of the sample by quartile of energy-adjusted SSB intake in childhood (visit one) are shown in Table 1. The mean age of participants at the childhood visit increased across SSB quartiles (mean age ± SD for highest vs. lowest SSB quartile: 10.7 ± 1.5 vs. 10.2 ± 1.5, respectively; *p* = 0.023). There were also differences in the racial/ethnic distribution across SSB quartiles (*p* < 0.001), whereby the highest quartile versus the lowest quartile had a higher percentage of Hispanic participants (52 vs. 29%, respectively), but fewer non-Hispanic white participants (33 vs. 60%, respectively).

### 2.2. Associations of SSB Intake in Childhood with Cardiometabolic Measures

The means and standard deviations for each cardiometabolic measure of interest at study visits in childhood and adolescence are shown in Table S1; generally, mean values for each cardiometabolic measure increased from childhood to adolescence, except for HDL cholesterol, which decreased. In linear mixed models adjusted for age, sex, and race/ethnicity, SSB intake in childhood was associated with higher fasting triglycerides across childhood/adolescence (β (95% CI) for highest versus lowest SSB intake quartile: 8.1 (−0.9, 17.0) mg/dL; p-trend = 0.057; Table 2). There were no notable associations between SSB intake in childhood and the other cardiometabolic risk factors (fasting glucose, fasting insulin, HOMA-IR, HDL cholesterol, or systolic blood pressure; Table 2). Thus, downstream analyses focused on triglycerides as the primary SSB-associated cardiometabolic outcome of interest.

### 2.3. Associations of SSB Intake in Childhood with Plasma Metabolites

A total of 180 metabolites (out of 767 metabolites measured by the untargeted metabolomics assay) were selected in LASSO regression with log-transformed SSB intake as the dependent variable, whereby selection was defined as having a non-zero coefficient in ≥40% of the bootstrap samples, which was a data-driven threshold described in more detail in the Methods section under “Statistical Analyses”. Among these selected metabolites, 136 (76%) had confirmed identities. The metabolites selected in LASSO regression with SSB intake are summarized in Table 3, which also reports the number of times that the metabolite was selected across bootstrap samples (whereby, “selection” was defined as having a non-zero regression coefficient) and its average β-coefficient across bootstrap samples. The selected metabolites were primarily lipids (45 metabolites, 25%), amino acid-related (44 metabolites, 24%), or xenobiotics (21 metabolites, 12%).

### 2.4. Associations of SSB-Related Metabolites in Childhood with Triglycerides

In Table 4, we show the subset of SSB-associated metabolites (from step two, reported in Table 3) that were also associated with fasting triglycerides (the primary cardiometabolic outcome of interest from step one/Table 2) across childhood and adolescence in linear mixed models adjusted for age, sex, and race/ethnicity. Of the 180 metabolites associated with SSB intake in childhood, 13 metabolites were also associated with triglycerides across childhood and adolescence in the same direction as their association with SSB intake in childhood, based on a Bonferroni-adjusted *p* < 0.00028 (α = 0.05/180 metabolites; Table 4). Among these 13 metabolites, 11 metabolites were lipid metabolites, while the remaining 2 were unknown metabolites (Table 4). The identified metabolites included four dicarboxylated fatty acids (chain lengths ranging 10 to 16), a lactosylceramide, and a plasmalogen, which were inversely associated with triglycerides (Table 4). There were also several mono- and diacylglycerols and phospholipids, which were positively associated with triglycerides (Table 4). Scatter plots visualizing the relationship between these lipid metabolites in childhood and triglycerides across childhood and adolescence are shown in Figure S1. Table S2 summarizes the full results from this step, i.e., associations between all 180 SSB-associated metabolites in childhood and triglycerides across childhood and adolescence, based on linear mixed models.

### 2.5. Sensitivity Analyses

In sensitivity analyses, we observed similar findings when we additionally adjusted each step for pubertal stage, which may be a mediator of the relationship between SSB intake and the cardiometabolic outcomes. Specifically, in step one, the association between SSB intake in childhood and triglycerides across childhood and adolescence was similar in magnitude and direction (β (95% CI): 7.4 (−1.5, 16.4) mg/dL for the highest versus lowest quartile of SSB intake; *p*-trend = 0.075). In step two, the LASSO regression selected a similar set of metabolites that were associated with SSB intake in childhood (170 metabolites were selected in ≥40% bootstrap samples, among which 153 (90%) were also selected in LASSO regression before adjusting for pubertal stage). In step three, the subset of SSB-associated metabolites from step two that were also associated with triglycerides across childhood and adolescence consisted of 11 of the 13 metabolites selected in the primary analysis above without adjusting for pubertal stage (two of the dicarboxylated fatty acids were not selected), which are summarized in Table S3.

## 3. Discussion

In this longitudinal cohort of diverse youth, we integrated data on diet, untargeted metabolomics, and conventional cardiometabolic risk factors using a “meet in the middle” approach to identify plasma metabolite alterations that link SSB intake in childhood with cardiometabolic risk across childhood adolescence. First, we found that energy-adjusted SSB intake was associated with higher fasting triglycerides across childhood and adolescence in this sample, but no other cardiometabolic risk factors (glucose, insulin, HOMA-IR, HDL cholesterol, or systolic blood pressure). Subsequently, we used robust selection criteria to identify 13 plasma metabolites that were associated both with SSB intake in childhood and with triglycerides across childhood and adolescence with the same directionality. All the metabolites that could be identified (11 of 13) were lipid-related metabolites, particularly dicarboxylated fatty acids, mono- and diacylglycerols, and phospholipids. This pattern of metabolite alterations may, therefore, reflect disruptions in lipid metabolism that causally link higher SSB intake in childhood with dyslipidemia risk, which will need to be investigated in future studies.

In the first step of the analysis, we found that childhood SSB intake was associated with higher triglycerides across childhood and adolescence, consistent with the literature [21]. This effect may be due to unregulated hepatic fructose metabolism [22], which can lead to hepatic substrate overload and increased hepatic de novo lipogenesis [23,24]. Fructose intake may also alter hepatic lipid metabolism by stimulating lipogenic gene expression via the activation of several transcriptional activator families, including carbohydrate-responsive element-binding protein (ChREBP), sterol regulatory element-binding protein (SREBP), and peroxisome proliferator-activated receptor (PPAR) [23,24], as well as indirectly by inhibiting fat oxidation [25]. Over time, these metabolic alterations contribute to intrahepatic lipid accumulation, which can promote a compensatory increase in the production and secretion of very-low-density lipoproteins (VLDLs), leading to higher plasma triglycerides, as we observed in this study. It was unexpected that we did not find significant associations of SSB intake in childhood with any of the other cardiometabolic risk factors. However, this aligns with the findings from a few other studies of relatively healthy populations, which also found a preferential association of SSB intake with lipids, but not with other markers of glucose-insulin homeostasis [26]. It is possible that disruptions in fasting triglycerides are a ‘first step’ in the metabolic milieu associated with SSB intake and that more prolonged exposure is needed to observe other associations, particularly among otherwise healthy youth.

After evaluating prospective associations between SSB intake and cardiometabolic outcomes, we identified the 180 plasma metabolites that were most strongly associated with SSB intake at the childhood visit using LASSO regression. LASSO is a data-driven, multivariate analytical technique that is ideally suited for dimension reduction and feature selection. These 180 metabolites were primarily related to lipid, amino acid, and xenobiotic metabolism pathways. In comparing the findings of the present study with published literature, we noted that one metabolite selected by LASSO, taurine, was one of the four urinary metabolites identified as a biomarker of SSB intake in a study of adults in Ireland by Gibbons et al. [18]. This consistent association may be because taurine is often an added ingredient (along with caffeine) in sugar-sweetened energy drinks, although, it is worth noting that circulating taurine may also be influenced by other food sources, such as meat and seafood, as well as endogenous taurine biosynthesis via methionine and cysteine metabolism [27]. The other three metabolites on the panel selected by Gibbons et al. (formate, isocitrate, and citrulline) were not selected by LASSO regression in this study, which may be due to differences in the biospecimen analyzed (i.e., urine versus blood) or the age range of the participants (i.e., adults versus children). We also note that none of the metabolites associated with SSB intake in this study overlapped with the metabolites associated with SSB intake in a similar analysis by Perng et al. based on the ELEMENT Project in Mexico City [20]. This may be due to differences in the geography of each cohort (including the composition and overall intake patterns of SSBs in the U.S. versus Mexico), as well as the untargeted metabolomics platforms employed (Metabolon in EPOCH vs. University of Michigan’s Metabolomics Research Core in ELEMENT) and the nuances of the analytical strategies used in each study. Collectively, this highlights the challenges in identifying dietary biomarkers that are consistent across different populations.

In the final step of our analysis, we filtered the 180 metabolites associated with SSB intake in childhood to 13 metabolites that were also associated with triglycerides across childhood and adolescence, which was the primary cardiometabolic outcome of interest. Except for two unknowns, all of these metabolites were identified as lipids. Specifically, four metabolites were dicarboxylated fatty acids, which are fatty acids with two carboxyl groups that can be generated from ω-oxidation or plant/vegetable intake [28] and have been shown to be markedly lower in children with obesity versus controls [29]. In this study, we also found inverse associations of dicarboxylated fatty acids with SSB intake and triglycerides, potentially reflecting disruptions in fatty acid catabolism/oxidation—a plausible metabolic disturbance that could be mediated by fructose-induced alterations in PPAR–alpha activity [30] and, in turn, contribute to elevated triglycerides. Other identified metabolites were two monoacylglycerols and one diacylglycerol, which were positively associated with SSB intake and triglycerides. This finding corroborates other studies showing that the accumulation of these lipid intermediates is associated with poorer cardiometabolic health [31,32], possibly via disruptions in insulin signaling and mitochondrial dysfunction [33,34], and, further, that their plasma levels were responsive to a healthy dietary pattern (Mediterranean diet) intervention [31].

Other lipid metabolites identified in this final step of our analysis included three glycerophospholipids and one lactosylceramide, a type of sphingolipid. Phospholipid alterations assessed by metabolomics or lipidomics have been consistently associated with cardiometabolic diseases, including diabetes/prediabetes and cardiovascular disease [35,36]. One of the phospholipids identified was 1-stearoyl-2-oleoyl-GPC (18:0/18:1), a common phosphatidylcholine (PC) found in animal membranes that was positively associated with SSBs and triglycerides in this study. This aligns with findings from a dietary intervention in adults showing that the same PC was directly associated with changes in triglycerides following an 8-week low-calorie diet [37]. The potential mechanisms underlying these associations warrant further investigation but may reflect the close link between hepatic phospholipid metabolism and triglyceride packaging and secretion from the liver [38].

A limitation was our reliance on a self-reported dietary assessment to quantify SSB intake, which can be prone to several biases (recall bias, social desirability bias, etc.), especially in children/adolescents with obesity [39]. However, because we assessed the relationship between dietary intake and the cardiometabolic outcomes prospectively, it is less likely that any reporting bias was differential with respect to the outcome. Moreover, we adjusted SSB intake for total energy intake for analyses, which may mitigate measurement error and improve the precision of estimates [40]. The metabolomics assay used was semi-quantitative in nature; thus, additional, targeted assays would be needed to assess absolute concentrations for metabolites. In addition, the sample was from one geographic region (Colorado, USA), which may limit the generalizability of our findings. Strengths of this study include the longitudinal study design, which allowed us to assess prospective associations between SSB intake and metabolome alterations in childhood with repeated measures of cardiometabolic risk, and the use of a comprehensive untargeted metabolomics profiling technique combined with a multivariate method (LASSO regression) with proven utility to protect against false positive findings for variable selection/dimension reduction. Additionally, the extensive laboratory, metabolic, anthropometric, and behavioral assessments performed on the EPOCH cohort allowed us to adjust for various potential confounding factors, and the relatively large sample size (~600 participants), especially compared to other metabolomics datasets in youth cohorts, provided statistical power to assess potential effect modification by sex.

### Conclusions

In a longitudinal, multiethnic cohort of children based in Colorado, we identified 13 metabolites, 11 of which were involved in lipid metabolic pathways, that link the prospective relationship between SSB intake in childhood with fasting triglyceride levels across childhood and adolescence. These intermediary lipid metabolites may not only represent potential biomarkers of higher SSB intake in youth but also may reflect underlying metabolic disruptions that are causally involved in the adverse effects of SSB intake on plasma triglycerides, supporting their prioritization in future investigations. Specifically, in addition to other prospective studies validating the utility of these metabolites as SSB biomarkers, experimental studies are needed to further understand the potential mechanisms underlying this interplay between SSB intake, lipid metabolite disruptions, and dyslipidemia in youth. This study also adds to the growing body of literature supporting a link between SSB intake and cardiometabolic abnormalities in youth, which supports the importance of more research aiming to identify and target factors that may influence childhood SSB intake, such as parental/family-, school/community-, or policy-related factors [41,42,43].

## 4. Materials and Methods

### 4.1. Study Population

This study included participants from the EPOCH study, a longitudinal, multiethnic cohort of youth in Colorado. The original aim was to examine the health effects on offspring of in utero exposure to maternal gestational diabetes mellitus (GDM) [44]. Participants were the offspring of mothers who were members of the Kaiser Permanente of Colorado (KPCO) health plan. We enrolled children who were exposed to maternal diabetes in utero (*n* = 99) and a random sample of children who were not exposed to maternal diabetes (*n* = 505). The first research visit occurred from 2006 to 2009, when offspring were 6–14 years old (median 10.6 years; “visit 1”). Among these participants, 413 returned for a second visit from 2012 to 2015 when offspring were 12–19 years old (median 16.8 years; “visit 2”). Mothers provided written informed consent and children participants provided written assent. This study was approved by the Colorado Multiple Institutional Review Board (Protocol no. 05-0623).

**Figure 2:**
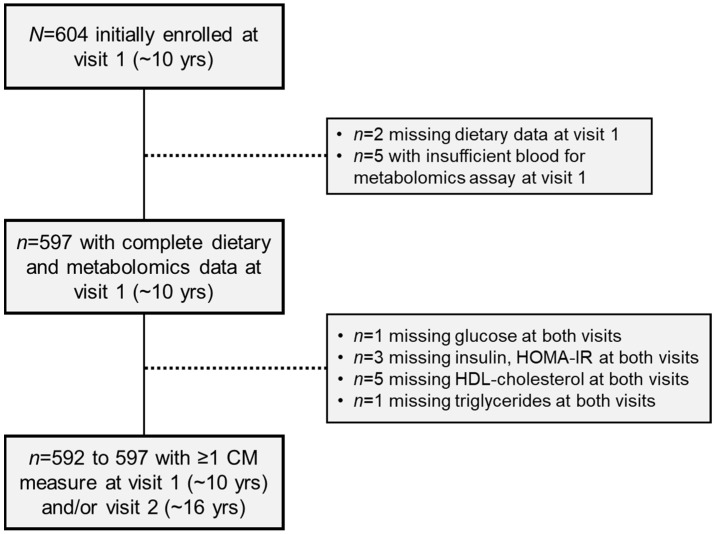
Selection of study participants from the Exploring Perinatal Outcomes among CHildren (EPOCH) Study. Abbreviations: CM, cardiometabolic; HOMA-IR, homeostatic model of assessment-insulin resistance; HDL, high-density lipoprotein.

For this analysis, we excluded two participants who were missing dietary data at visit 1 and five participants with insufficient blood volume for metabolomics analysis at visit 1, resulting in an analytical sample of 597 participants. In analyses examining the prospective associations with cardiometabolic risk factors across childhood and adolescence, we also excluded the following participants who were missing data *both* at visit 1 and visit 2 for the following measures (i.e., no data at either visit for the outcome of interest): *n* = 1 missing glucose at both visits; *n* = 3 missing insulin and HOMA-IR at both visits; *n* = 5 missing HDL cholesterol at both visits; *n* = 1 missing triglycerides at both visits. Participant selection is summarized in Figure 2.

### 4.2. Dietary Assessment

Dietary intake was assessed at both visits using a modified version of the Block Kids Food Frequency Questionnaire [45], a semi-quantitative food frequency questionnaire that has been validated in children as young as 8 years old [46,47]. The questionnaire queried whether the participant consumed each food/beverage item in the past week and, if so, how many days per week (ranging from ‘one day’ to ‘every day’) and the usual amount eaten per day. The full questionnaire may be available upon reasonable request. Completed questionnaires were then analyzed to estimate individual intakes of total energy, nutrients, and food groups per day. For each participant, SSB intake in childhood was assessed by summing the servings per day from the following: sodas, fruit drinks (i.e., Sunny Delight, Hawaiian Punch, etc.), sports drinks (i.e., Gatorade), and sweetened tea or coffee. We used the residual method to adjust SSB intake for total energy intake per day [48]. Participants were then grouped based on their quartile of energy-adjusted SSB intake for later analyses.

### 4.3. Untargeted Metabolomics Profiling of Plasma

Fasting blood samples were collected from all participants at both visits by trained phlebotomists. All samples were refrigerated immediately, processed within 24 h, and stored at −80 °C until the time of analysis. As previously described [49,50], untargeted metabolomics profiling was performed on stored fasting plasma samples by Metabolon using a multiplatform mass spectroscopy-based technique, which identified 1193 unique features at both time points. Prior to formal statistical analysis, we removed metabolites with ≥20% missing values per batch. The remaining missing values were imputed using the K-nearest neighbor algorithm (with K = 10) [51]. The samples were analyzed in two batches: the first batch of participants had 913 metabolites after removing those with high missingness, and the second batch had 898 metabolites. We then merged the two batches for subsequent data processing and retained 767 metabolites that were present in both batches. The retained metabolites then underwent log_10_-transformation, followed by metabolite normalization and correction for batch effects (as well as other biological and technical variability) using the remove unwanted variation method (with K = 2 factors of unwanted variation estimated from the data) [52]. All the above preprocessing steps were performed using R Statistical Software (version 3.5.3) [53].

### 4.4. Cardiometabolic Risk Assessments

We used fasting blood samples from both visits to assess the following markers of cardiometabolic risk across childhood and adolescence. Fasting glucose was assessed enzymatically, and fasting insulin was assessed by using radioimmunoassay (Millipore, Darmstadt, Germany). Fasting glucose and insulin were then used to estimate insulin resistance using the homeostatic model of assessment (HOMA-IR) [54] Fasting blood lipids, including HDL cholesterol and triglycerides, were assayed on the Olympus AU400 advanced chemistry analyzer system. At both visits in childhood and adolescence, participants’ blood pressure was also measured twice in the sitting position using an oscillometric monitor (Dinamap ProCare V100). For this analysis, we used the average of the two values and focused on systolic blood pressure only.

### 4.5. Other Covariate Assessments

Exposure to maternal GDM during pregnancy was ascertained from the KPCO perinatal database and defined as a physician’s diagnosis of gestational diabetes based on routine screening at 24–28 weeks of gestation using the standard two-step protocol [55]. Child race/ethnicity was self-reported at visit 1 as being non-Hispanic white, non-Hispanic black, Hispanic, or non-Hispanic other. The child’s height (kg) and weight (cm) were assessed at both visits by trained research staff while wearing light clothing and without shoes. These measurements were used to calculate age- and sex-specific body mass index (BMI) *z*-scores using the WHO growth reference for children aged 5–19 years [56]. At both visits, participants reported their pubertal development based on pictorial diagrams of the Tanner stages, which had been validated against physician-assessed Tanner staging and puberty-related hormones [57], and which bases pubertal stage on pubic hair development in boys and breast development in girls. Participants were then categorized as pre-pubertal (Tanner = 1), pubertal (Tanner = 2 or 3), and late/post-pubertal (Tanner = 4 or 5).

### 4.6. Statistical Analyses

We first examined bivariate associations between energy-adjusted SSB intake quartiles in childhood and the background characteristics of the sample in childhood. For categorical variables, we reported counts and frequencies for each sub-category according to SSB intake quartile and tested differences across quartiles using Chi-squared tests. For continuous variables, we reported means and standard deviations according to SSB quartile and tested for differences across SSB quartiles using a one-way analysis of variance (ANOVA).

Next, a “meet in the middle” approach was employed to identify the plasma metabolites that may mark the relationship between SSB intake and cardiometabolic risk. In the first step, we examined the associations of SSB quartiles in childhood (visit 1) with repeated measures of cardiometabolic risk (fasting glucose, fasting insulin, HOMA-IR, HDL cholesterol, fasting triglycerides, and systolic blood pressure) across childhood and adolescence (visit 1 and visit 2) using mixed-effects models adjusted for child age, sex, and race/ethnicity. Models included a random intercept for each participant ID to account for intra-individual correlations in cardiometabolic measures across visits and an unstructured covariance matrix. We assessed effect modification by sex using product interaction terms, but none reached significance (all *p* > 0.10); therefore, all analyses were conducted on the entire sample. Results are reported as regression coefficients and 95% confidence intervals (CIs) for the 2nd, 3rd, and 4th quartiles of SSB intake when compared to the 1st quartile (the reference). We also tested for a linear trend across quartiles using a continuous variable based on the median value for each SSB quartile. For this step, we considered cardiometabolic risk factors for further exploration in downstream analyses if *p* < 0.10 for a linear trend across SSB quartiles.

In the second step, we identified plasma metabolites that were cross-sectionally associated with SSB intake in childhood (visit 1) by employing LASSO regression [58], using the *glmnet* package in R [53]. Briefly, LASSO is a regularized regression technique designed to select the strongest variables associated with an outcome of interest from a high-dimensional and correlated set of predictors. This is done by imposing a tuning parameter on the model that shrinks the regression coefficients for weaker variables to zero during feature selection, thereby removing weak but statistically significant associations that may represent false positive findings. Ten-fold cross validation was used to determine the tuning parameter that achieved the minimum mean error. To perform more stabilizing of variable selection, instead of running once, LASSO regression was carried out with 100 bootstrap samples with log-transformed SSB intake in childhood as the dependent variable and with adjustments for child age, sex, and race/ethnicity, as unpenalized variables. Metabolites were considered for downstream analyses if they were selected by LASSO (i.e., non-zero coefficient) in ≥40% of bootstrap samples. This threshold was determined by firstly calculating the number of metabolites selected, on average, across the 100 bootstrap iterations (202 metabolites selected on average). We then calculated the number of metabolites selected per 5-unit threshold increment and chose a threshold that selected a number of metabolites closest to the average from bootstrapping (180 metabolites were selected with a threshold of ≥40%).

In the third step, we investigated whether SSB-associated metabolites in childhood (selected from step 2) were also associated with SSB-associated cardiometabolic risk factors across childhood and adolescence (selected from step 1) using a second set of linear mixed-effects models with each metabolite as the independent variable and the repeated measures of each cardiometabolic measure across childhood and adolescence used as the dependent variable. Models were again adjusted for age, sex, and race/ethnicity, and included a random intercept for each participant’s ID and an unstructured covariance matrix. Results are reported as regression coefficients and 95% CIs for the effect of a 1 unit increase in each metabolite on cardiometabolic measures. Metabolites were selected as being intermediary biomarkers if the regression coefficient’s *p*-value was below a Bonferroni-adjusted threshold (α = 0.05/number of metabolites tested) and if the direction of the association with the cardiometabolic measure was the same as with the SSB intake. We also performed a sensitivity analysis where we additionally adjusted each step of the analysis for pubertal stage (pre-pubertal, pubertal, or late-pubertal), which can also affect cardiometabolic status during childhood [59]. Unless otherwise stated, statistical analyses were performed using SAS Statistical Software (version 9.4; Cary, NC, USA).

## Figures and Tables

**Table 1:** Descriptive statistics of 597 EPOCH participants during childhood according to sugar sweetened beverage (SSB) intake.

	SSB Intake in Childhood ^1^:				
	Quartile 1 (0 to 0.25 Servings/d)	Quartile 2 (0.26 to 0.54 Servings/d)	Quartile 3 (0.55 to 1.00 Servings/d)	Quartile 4 (1.01 to 5.12 Servings/d)	
Variable:	Mean (SD) or Count (%)	Mean (SD) or Count (%)	Mean (SD) or Count (%)	Mean (SD) or Count (%)	*p*-Value ^2^
N	148	149	150	150	
Age (years), mean (SD)	10.2 (1.5)	10.3 (1.4)	10.5 (1.4)	10.7 (1.5)	0.023
Male Sex, *n* (%)	63 (43%)	72 (48%)	75 (50%)	87 (58%)	0.064
Race/ethnicity, *n* (%):					<0.001
Hispanic	43 (29%)	52 (35%)	57 (38%)	78 (52%)	
NH White	89 (60%)	77 (52%)	71 (47%)	49 (33%)	
NH Black	10 (7%)	8 (5%)	13 (9%)	17 (11%)	
NH Other	6 (4%)	12 (8%)	9 (6%)	6 (4%)	
BMI z-score, mean (SD)	0.28 (1.30)	0.19 (1.22)	0.22 (1.21)	0.37 (1.21)	0.611
Energy intake (kcal/d), mean (SD)	1720 (501)	1808 (503)	1834 (527)	1810 (649)	0.292
Pubertal stage, *n* (%):					0.793
Pre-pubertal (Tanner = 1)	69 (49%)	68 (48%)	67 (47%)	69 (49%)	
Pubertal (Tanner = 2 or 3)	71 (51%)	74 (52%)	75 (53%)	72 (51%)	
Late/post-pubertal (Tanner = 4)	0 (0%)	1 (1%)	0 (0%)	0 (0%)	
In utero GDM Exposure, *n* (%)	29 (20%)	22 (15%)	23 (15%)	25 (16%)	0.683
SSB intake ^3^ (serving/d), mean (SD)	0.11 (0.09)	0.39 (0.08)	0.77 (0.13)	1.86 (0.91)	

^1^ SSB intake quartiles were determined based on energy adjusted SSB intake using the residual method. ^2^ Differences in each child’s characteristics by SSB intake quartile were assessed using analysis of variance (ANOVA) for continuous characteristics and Chi-squared tests for categorical characteristics. ^3^ SSB intake was energy-adjusted using the residual method. Abbreviations: SSB, sugar sweetened beverages; BMI, body mass index; GDM, gestational diabetes mellitus.

**Table 2:** Prospective associations between sugar sweetened beverage (SSB) intake in childhood and cardiometabolic risk factors across childhood and adolescence.

	SSB Intake in Childhood ^1^:			
	Quartile 2 vs. 1	Quartile 3 vs. 1	Quartile 4 vs. 1	Linear Trend
Outcome:	β (95% CI) ^2^	β (95% CI) ^2^	β (95% CI) ^2^	*p*-Value ^2,3^
Glucose (mg/dL)	−0.7 (−4.1, 2.7)	−0.7 (−4.2, 2.7)	−1.4 (−4.9, 2.2)	0.488
Insulin (μIU/mL)	−1.7 (−3.5, 0.1)	−1.1 (−2.9, 0.7)	−1.3 (−3.1, 0.6)	0.426
HOMA-IR	−0.7 (−1.3, −0.1)	−0.4 (−1.0, 0.2)	−0.5 (−1.1, 0.1)	0.330
HDL Cholesterol (mg/dL)	−0.6 (−2.7, 1.6)	−0.8 (−3.0, 1.4)	−0.1 (−2.4, 2.1)	0.973
Triglycerides (mg/dL)	1.6 (−7.0, 10.3)	4.9 (−3.8, 13.6)	8.1 (−0.9, 17.0)	0.057
Systolic blood pressure (mm Hg)	−1.8 (−3.7, 0.2)	−1.0 (−3.0, 1.0)	−0.9 (−2.9, 1.1)	0.770

^1^ SSB intake quartiles were determined based on energy-adjusted SSB intake using the residual method. ^2^ Estimates based on mixed-effects models adjusted for participant age across visits, sex, and race/ethnicity (Hispanic, non-Hispanic white, non-Hispanic black, or non-Hispanic other). All models included a participant-specific random intercept. ^3^ Linear trends across quartiles were assessed using the median value for each quartile. Abbreviations: HOMA-IR, homeostatic model of assessment for insulin resistance; HDL, high-density lipoprotein.

**Table 3:** Metabolite features in childhood associated with sugar sweetened beverage intake in childhood, based on least absolute shrinkage and selection operator (LASSO) regression.

Metabolite Name ^1^	Pathway	Sub-Pathway	Average β ^2^	Count ^3^
Beta-citrylglutamate	Amino acid	Glutamate metabolism	0.140	61
Carboxyethyl-GABA	Amino Acid	Glutamate Metabolism	0.113	52
N-acetyl-aspartyl-glutamate (NAAG)	Amino Acid	Glutamate Metabolism	0.108	56
N-acetylglutamate	Amino Acid	Glutamate Metabolism	−0.110	52
Pyroglutamine *	Amino acid	Glutamate metabolism	0.072	50
Cys-gly, oxidized	Amino acid	Glutathione metabolism	0.222	84
Betaine	Amino acid	Glycine/serine/threonine metabolism	0.212	44
Dimethylglycine	Amino acid	Glycine/serine/threonine metabolism	−0.187	55
Sarcosine	Amino acid	Glycine/serine/threonine metabolism	0.147	58
Threonine	Amino acid	Glycine/serine/threonine metabolism	−0.153	45
3-methylhistidine	Amino acid	Histidine metabolism	0.026	43
N-acetylhistidine	Amino Acid	Histidine metabolism	0.434	89
Trans-urocanate	Amino acid	Histidine metabolism	0.065	50
2,3-dihydroxy-2-methylbutyrate	Amino acid	BCAA metabolism	0.049	44
3-hydroxy-2-ethylpropionate	Amino acid	BCAA metabolism	−0.196	50
3-hydroxyisobutyrate	Amino acid	BCAA metabolism	0.042	40
3-methyl-2-oxobutyrate	Amino acid	BCAA metabolism	0.032	43
3-methylglutaconate	Amino acid	BCAA metabolism	−0.136	53
Alpha-hydroxyisocaproate	Amino acid	BCAA metabolism	−0.197	59
Isoleucine	Amino acid	BCAA metabolism	0.338	45
Isovalerylcarnitine (C5)	Amino Acid	BCAA metabolism	0.157	63
Isovalerylglycine	Amino acid	BCAA metabolism	0.096	51
5-(galactosylhydroxy)-L-lysine	Amino Acid	Lysine Metabolism	−0.094	40
5-hydroxylysine	Amino acid	Lysine metabolism	0.133	62
Glutarylcarnitine (C5-DC)	Amino Acid	Lysine Metabolism	−0.280	89
N,N,N-trimethyl-5-aminovalerate	Amino Acid	Lysine Metabolism	0.157	55
N6-acetyllysine	Amino Acid	Lysine Metabolism	−0.165	41
Cysteine s-sulfate	Amino acid	Methionine/cysteine/SAM metabolism	0.124	68
Methionine sulfone	Amino acid	Methionine/cysteine/SAM metabolism	0.071	42
Taurine	Amino acid	Methionine/cysteine/SAM metabolism	0.165	57
(N(1) + N(8))-acetylspermidine	Amino Acid	Polyamine Metabolism	0.122	44
4-acetamidobutanoate	Amino acid	Polyamine metabolism	−0.173	48
Indoleacetate	Amino acid	Tryptophan metabolism	0.066	53
Indolepropionate	Amino acid	Tryptophan metabolism	−0.057	52
Tryptophan betaine	Amino acid	Tryptophan metabolism	−0.045	62
3-methoxytyrosine	Amino acid	Tyrosine metabolism	0.268	71
N-acetyltyrosine	Amino Acid	Tyrosine Metabolism	−0.116	53
P-cresol glucuronide *	Amino acid	Tyrosine metabolism	0.030	42
Phenol sulfate	Amino acid	Tyrosine metabolism	−0.003	40
Thyroxine	Amino acid	Tyrosine metabolism	0.090	42
Tyramine O-sulfate	Amino Acid	Tyrosine Metabolism	0.210	100
Argininate *	Amino acid	Urea cycle; arginine/proline metabolism	−0.094	57
N-acetylarginine	Amino Acid	Urea cycle; arginine/proline metabolism	−0.121	43
N-methylproline	Amino Acid	Urea cycle; arginine/proline metabolism	−0.029	40
N-acetylglucosamine/galactosamine	Carbohydrate	Amino sugar Metabolism	−0.136	42
Mannitol/sorbitol	Carbohydrate	Hexose metabolism	−0.045	49
Arabitol/xylitol	Carbohydrate	Pentose metabolism	−0.230	62
Arabonate/xylonate	Carbohydrate	Pentose metabolism	−0.096	40
Ribonate	Carbohydrate	Pentose metabolism	−0.094	41
Ribulonate/xylulonate *	Carbohydrate	Pentose metabolism	0.121	54
Gulonate *	Cofactors	Ascorbate/aldarate metabolism	0.144	55
Threonate	Cofactors	Ascorbate/aldarate metabolism	0.047	45
1-methylnicotinamide	Cofactors	Nicotinate/nicotinamide metabolism	0.352	92
Quinolinate	Cofactors	Nicotinate/nicotinamide metabolism	0.070	44
Trigonelline (N’-methylnicotinate)	Cofactors	Nicotinate/Nicotinamide Metabolism	−0.058	58
Pantothenate	Cofactors	Pantothenate/CoA metabolism	−0.115	40
Beta-cryptoxanthin	Cofactors	Vitamin A metabolism	−0.047	50
Carotene diol (2)	Cofactors	Vitamin A metabolism	0.110	41
Retinol (Vitamin A)	Cofactors	Vitamin A Metabolism	−0.239	61
Pyridoxate	Cofactors	Vitamin B6 metabolism	−0.084	47
Deoxycarnitine	Lipid	Carnitine metabolism	−0.129	46
Ceramide (d18:1/14:0, d16:1/16:0) *	Lipid	Ceramides	0.115	50
N-stearoyl-sphingadienine (d18:2/18:0) *	Lipid	Ceramides	−0.289	86
Palmitoyl-arachidonoyl-glycerol (36:4) *	Lipid	Diacylglycerol	−0.067	45
Palmitoyl-linoleoyl-glycerol (16:0/18:2) *	Lipid	Diacylglycerol	0.096	44
Stearoyl-arachidonoyl-glycerol (18:0/20:4) *	Lipid	Diacylglycerol	−0.079	41
N-oleoylserine	Lipid	Endocannabinoid	0.227	61
Adipoylcarnitine (C6-DC)	Lipid	Fatty Acid Metabolism (Acyl Carnitine)	0.085	55
Laurylcarnitine (C12)	Lipid	Fatty Acid Metabolism (Acyl Carnitine)	0.124	44
Linolenoylcarnitine (C18:3) *	Lipid	Fatty Acid Metabolism (Acyl Carnitine)	0.083	42
3-hydroxybutyroylglycine *	Lipid	Fatty acid metabolism (Acyl Glycine)	−0.066	50
N-palmitoylglycine	Lipid	Fatty Acid Metabolism (Acyl Glycine)	−0.187	52
Hexadecanedioate (C16-DC)	Lipid	Fatty Acid/Dicarboxylate	−0.124	50
Hexadecenedioate (C16:1-DC) *	Lipid	Fatty Acid/Dicarboxylate	−0.252	65
Octadecadienedioate (C18:2-DC) *	Lipid	Fatty Acid/Dicarboxylate	0.107	67
Sebacate (C10-DC)	Lipid	Fatty Acid/Dicarboxylate	−0.063	41
Tetradecanedioate (C14-DC)	Lipid	Fatty Acid/Dicarboxylate	−0.317	77
12,13-dihome	Lipid	Fatty Acid/Dihydroxy	0.063	41
2-hydroxylaurate	Lipid	Fatty acid/monohydroxy	0.303	78
2-hydroxynervonate *	Lipid	Fatty acid/monohydroxy	0.174	56
Glycosyl ceramide (d38:1) *	Lipid	Hexosylceramides (HCER)	0.339	79
Glycosyl-N-stearoyl-sphingosine (d36:1)	Lipid	Hexosylceramides (HCER)	0.160	43
Lactosyl-N-behenoyl-sphingosine (d40:1) *	Lipid	Lactosylceramides (LCER)	−0.230	84
Arachidate (20:0)	Lipid	Long chain fatty acid	0.127	48
Margarate (17:0)	Lipid	Long chain fatty acid	0.217	44
1-linoleoyl-GPG (18:2) *	Lipid	Lysophospholipid	0.091	41
1-arachidonylglycerol (20:4)	Lipid	Monoacylglycerol	0.108	58
1-linolenoylglycerol (18:3)	Lipid	Monoacylglycerol	0.182	84
1-oleoylglycerol (18:1)	Lipid	Monoacylglycerol	0.105	47
2-arachidonoylglycerol (20:4)	Lipid	Monoacylglycerol	0.083	56
1-myristoyl-2-arachidonoyl-GPC (34:4) *	Lipid	Phosphatidylcholine (PC)	−0.122	42
1-stearoyl-2-oleoyl-GPC (18:0/18:1)	Lipid	Phosphatidylcholine (PC)	0.440	64
1,2-dipalmitoyl-GPC (16:0/16:0)	Lipid	Phosphatidylcholine (PC)	0.466	57
1-palmitoyl-2-arachidonoyl-GPI (36:4) *	Lipid	Phosphatidylinositol (PI)	0.199	66
1-(1-enyl-palmitoyl)-2-arachidonoyl-GPC (P-36:4) *	Lipid	Plasmalogen	0.166	44
1-(1-enyl-palmitoyl)-2-palmitoleoyl-GPC (P-32:1) *	Lipid	Plasmalogen	−0.173	64
Adrenate (22:4n6)	Lipid	Polyunsaturated fatty acid (n3/n6)	0.117	52
Glycochenodeoxycholate	Lipid	Primary bile acid metabolism	−0.035	41
Taurocholate	Lipid	Primary bile acid metabolism	0.057	54
Glycolithocholate sulfate *	Lipid	Secondary bile acid metabolism	−0.044	45
Lithocholate sulfate (1)	Lipid	Secondary bile acid metabolism	−0.037	42
Sphinganine-1-phosphate	Lipid	Sphingolipid synthesis	−0.147	63
Sphingomyelin (d43:1) *	Lipid	Sphingomyelins	−0.280	74
Sphingomyelin (d42:4) *	Lipid	Sphingomyelins	0.201	45
7-alpha-hydroxy-3-oxo-4-cholestenoate	Lipid	Sterol	−0.082	42
Allantoin	Nucleotide	Purine metabolism: xanthine/inosine	−0.376	80
Adenine	Nucleotide	Purine metabolism: adenine	−0.147	42
Guanosine	Nucleotide	Purine metabolism: guanine	−0.145	97
N2,N2-dimethylguanosine	Nucleotide	Purine Metabolism: guanine	0.279	46
Orotate	Nucleotide	Pyrimidine metabolism: orotate	−0.238	73
Orotidine	Nucleotide	Pyrimidine metabolism: orotate	0.073	41
3-aminoisobutyrate	Nucleotide	Pyrimidine metabolism: thymine	0.059	46
5,6-dihydrothymine	Nucleotide	Pyrimidine metabolism: thymine	0.181	65
Leucylalanine	Peptide	Dipeptide	0.044	47
Gamma-glutamylcitrulline *	Peptide	Gamma-glutamyl amino acid	−0.056	42
3-methoxycatechol sulfate (1)	Xenobiotics	Benzoate metabolism	0.082	81
3-phenylpropionate (hydrocinnamate)	Xenobiotics	Benzoate metabolism	−0.057	61
4-ethylphenylsulfate	Xenobiotics	Benzoate metabolism	0.027	48
4-hydroxyhippurate	Xenobiotics	Benzoate metabolism	0.087	52
4-methylguaiacol sulfate	Xenobiotics	Benzoate metabolism	0.041	40
Methyl-4-hydroxybenzoate sulfate	Xenobiotics	Benzoate metabolism	0.046	75
2-naphthol sulfate	Xenobiotics	Chemical	0.029	45
3-hydroxypyridine sulfate	Xenobiotics	Chemical	−0.042	45
Perfluorooctanoate (PFOA) *	Xenobiotics	Chemical	−0.141	62
Sulfate *	Xenobiotics	Chemical	0.336	56
Hydroquinone sulfate	Xenobiotics	Drug–topical agents	−0.185	92
2-isopropylmalate	Xenobiotics	Food component/plant	−0.064	47
2-piperidinone	Xenobiotics	Food component/plant	−0.040	52
2,3-dihydroxyisovalerate	Xenobiotics	Food component/plant	−0.050	52
3,4-methyleneheptanoate	Xenobiotics	Food component/plant	0.059	50
Ergothioneine	Xenobiotics	Food component/plant	−0.400	98
Erythritol	Xenobiotics	Food component/plant	0.110	51
Glucuronide of piperine metabolite *	Xenobiotics	Food Component/Plant	−0.039	45
Pyrraline	Xenobiotics	Food component/plant	−0.088	64
Thymol sulfate	Xenobiotics	Food component/plant	−0.030	59
5-acetylamino-6-amino-3-methyluracil	Xenobiotics	Xanthine metabolism	0.097	90

^1^ This table only shows the metabolites that were identified. A total of 44 unknown metabolites are not shown. ^2^ Average β-coefficients were calculated as the average of all regression coefficients for each metabolite across 100 bootstrap samples. ^3^ Indicates the number of times the metabolite was selected (based on having a non-zero coefficient) across 100 bootstrap samples. Metabolites shown here were selected in ≥40% of bootstrap samples. * Indicates tier 2 identification in which no commercially available authentic standard can be found; however, it was annotated based on accurate mass, spectral, and chromatographic similarity to tier 1-identified compounds.

**Table 4:** Prospective associations between the selected metabolites associated with sugar-sweetened beverage intake in childhood and fasting triglycerides across childhood and adolescence.

Metabolite Name ^1^	Pathway	Sub-Pathway	β (95% CI) ^2^	*p*-Value ^3^
Palmitoyl-linoleoyl-glycerol (16:0/18:2) *	Lipid	Diacylglycerol	78.3 (66.3, 90.2)	<1.00 × 10^−7^
Tetradecanedioate (C14-DC)	Lipid	Fatty Acid/Dicarboxylate	−35.9 (−51.2, −20.5)	3.79 × 10^−6^
Hexadecenedioate (C16:1-DC) *	Lipid	Fatty Acid/Dicarboxylate	−30.9 (−46.3, −15.6)	8.35 × 10^−5^
Hexadecanedioate (C16-DC)	Lipid	Fatty Acid/Dicarboxylate	−40.0 (−56.7, −23.2)	8.47 × 10^−6^
Sebacate (C10-DC)	Lipid	Fatty Acid/Dicarboxylate	−31.6 (−45.4, −17.8)	6.15 × 10^−6^
Lactosyl-N-behenoyl-sphingosine (d40:1) *	Lipid	Lactosylceramides (LCER)	−39.6 (−55.4, −23.8)	1.23 × 10^−6^
1-linolenoylglycerol (18:3)	Lipid	Monoacylglycerol	46.6 (35.6, 57.6)	<1.00 × 10^−7^
1-oleoylglycerol (18:1)	Lipid	Monoacylglycerol	88.3 (73.9, 102.7)	<1.00 × 10^−7^
1-stearoyl-2-oleoyl-GPC (18:0/18:1)	Lipid	Phosphatidylcholine (PC)	76.1 (47.7, 104.5)	2.26 × 10^−7^
1-palmitoyl-2-arachidonoyl-GPI (36:4) *	Lipid	Phosphatidylinositol (PI)	71.6 (56.6, 86.6)	<1.00 × 10^−7^
1-(1-enyl-palmitoyl)-2-palmitoleoyl-GPC (P-32:1) *	Lipid	Plasmalogen	−69.3 (−92.8, −45.7)	<1.00 × 10^−7^

^1^ This table only shows the 11 metabolites that were identified. A total of 2 unknown metabolites are not shown. ^2^ Estimates based on linear mixed-effects models adjusted for participant age across visits, sex, and race/ethnicity (Hispanic, non-Hispanic white, non-Hispanic black, or non-Hispanic other). All models included a participant-specific random intercept. ^3^ Displaying raw *p*-values from linear mixed effects models. Only metabolites with a *p*-value less than the Bonferroni-corrected *p* < 0.000278 (0.05/180 metabolite tests or 2.78 × 10^−4^) and with an estimate in the same direction as with SSB intake in childhood are shown. * Indicates tier 2 identification in which no commercially available authentic standard can be found; however, it was annotated based on accurate mass, spectral, and chromatographic similarity to tier 1 identified compounds.

## Data Availability

Data are available upon reasonable request.

## Supplementary Materials

The following supporting information can be downloaded at: https://www.mdpi.com/article/10.3390/metabo12060559/s1, Figure S1: Associations between plasma metabolites in childhood (selected from Table 4) with triglycerides across childhood and adolescence, Table S1: Means and standard deviations for the cardiometabolic measures of interest at each visit in childhood and adolescence, Table S2: Prospective associations between all 180 metabolites associated with sugar sweetened beverage intake in childhood (from step 2) and fasting triglycerides across childhood and adolescence, Table S3: Sensitivity Analysis—Prospective associations between select metabolites associated with sugar sweetened beverage intake in childhood and triglycerides across childhood and adolescence with additional adjustment for pubertal stage.
