# Supplementary material for: Metabolome Alterations Linking Sugar-Sweetened Beverage Intake with Dyslipidemia in Youth: The Exploring Perinatal Outcomes among CHildren (EPOCH) Study
Source: Metabolites. 2022 Jun 17;12(6):559. doi: 10.3390/metabo12060559 (PMC9228193; doi:10.3390/metabo12060559)
Supplement: Supplementary file 1 [file metabolites-12-00559-s001.zip › metabolites-1690822-supplementary.pdf]

Online Supplementary Materials

Title:

Metabolome Alterations Linking Sugar Sweetened Beverage (SSB) Intake with Dyslipidemia in Youth: The Exploring Perinatal Outcomes among Children (EPOCH) Study

Authors & Affiliations:

Catherine C. Cohen[1,2]; Dana Dabelea[1,2,3;] Katerina Kechris[4]; Gregory Michelotti[5]; Lu Tang[6]; Kartik Shankar[1,2]; Michael I. Goran[7]; Wei Perng[2,3,8].

[1] Department of Pediatrics, School of Medicine, University of Colorado Anschutz Medical Campus, Aurora, CO, USA.

[2] Lifecourse Epidemiology of Adiposity and Diabetes (LEAD) Center, University of Colorado Denver Anschutz Medical Campus, Aurora CO, USA.

[3] Department of Epidemiology, Colorado School of Public Health, University of Colorado Anschutz Medical Campus, Aurora, CO, USA.

[4] Department of Biostatistics and Informatics, Colorado School of Public Health, University of Colorado Anschutz Medical Campus, Aurora, CO, USA.

[5] Metabolon, Inc., Chapel Hill, NC, USA.

[6] Department of Biostatistics, University of Pittsburgh School of Public Health, Pittsburgh, PA, USA.

[7] Department of Population and Public Health Sciences, Keck School of Medicine, University of Southern California, Los Angeles, CA, USA.

[8] Department of Nutritional Sciences, University of Michigan School of Public Health, Ann Arbor, MI, USA.

Corresponding Author:

Catherine Cioffi Cohen, PhD, RDN; Catherine.Cioffi@cuanschutz.edu.

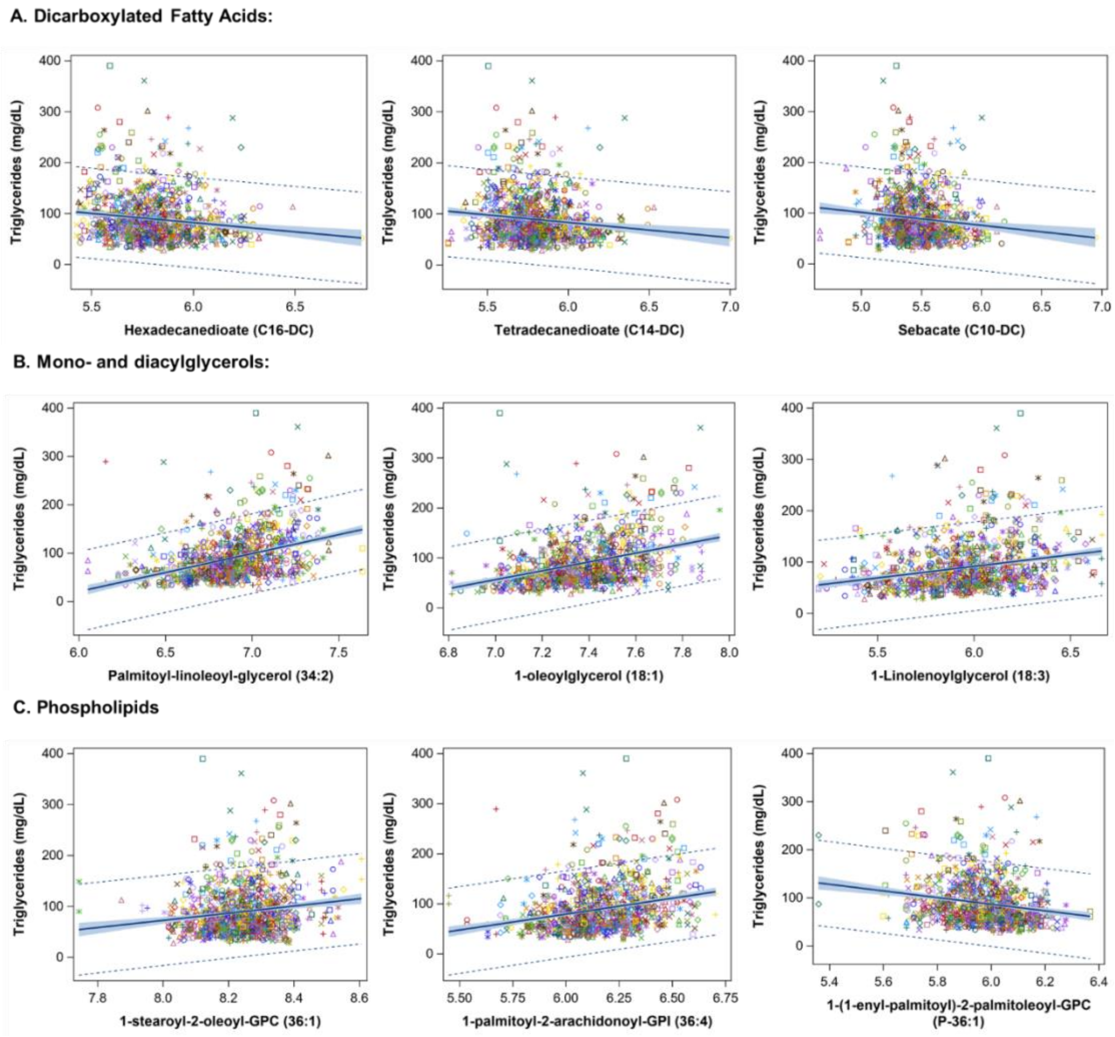

Figure S1. Scatter plots with regression lines showing associations between plasma metabolites in childhood (selected from Table 4) with triglycerides across childhood and adolescence. All associations were below the Bonferroni-adjusted threshold of $p<0.00028$. Each unique marker represents an individual participant. Two metabolites from Table 4 are not shown in this figure: hexadecanedioate (a dicarboxylated fatty acid) and lactosyl-N-behenoyl-sphingosine (a lactosylceramide).

Table S1. Means and standard deviations for the cardiometabolic measures of interest at each visit in childhood and adolescence.

	Childhood Visit (~10 years):		Adolescent Visit (~16 years)		Childhood vs. Adolescence
**Cardiometabolic Measure**	**N**	**Mean (SD)**	**N**	**Mean (SD)**	**P-difference[a]**
Glucose (mg/dL)	598	81.9 (12.9)	410	90.7 (23.4)	<0.001
Insulin (μIU/mL)	596	11.0 (8.1)	409	16.9 (11.3)	<0.001
HOMA-IR	596	2.2 (1.7)	409	4.0 (4.7)	<0.001
HDL-Cholesterol (mg/dL)	592	49.8 (11.6)	410	45.9 (9.7)	<0.001
Triglycerides (mg/dL)	598	89.9 (44.2)	410	87.5 (47.6)	0.417
Systolic blood pressure (mm Hg)	599	103.0 (10.0)	410	115.8 (10.8)	<0.001

[a] P-values were calculated using Student's t-test to assess differences in cardiometabolic measures between childhood and adolescence.

Table S2. Prospective associations between all 180 metabolites associated with sugar sweetened beverage intake in childhood (from step 2) and fasting triglycerides across childhood and adolescence.

Metabolite[a]	Pathway	β (95% CI)[b]	P-value[c]
beta-citrylglutamate	Amino Acid	-16.8 (-28.5, -5.1)	0.00505
carboxyethyl-GABA	Amino Acid	-22.2 (-47.6, 3.1)	0.08539
N-acetyl-aspartyl-glutamate (NAAG)	Amino Acid	-20 (-37, -2.9)	0.02172
N-acetylglutamate	Amino Acid	24 (1.2, 46.8)	0.03912
pyroglutamine*	Amino Acid	-1.1 (-16.3, 14.1)	0.88630
cys-gly, oxidized	Amino Acid	-12.5 (-23, -2)	0.01979
betaine	Amino Acid	-66.2 (-103.6, -28.8)	0.00055
dimethylglycine	Amino Acid	-3.7 (-31.7, 24.3)	0.79577
sarcosine	Amino Acid	-32.9 (-53.7, -12.1)	0.00199
threonine	Amino Acid	-0.0 (-33.8, 33.7)	0.99865
3-methylhistidine	Amino Acid	-1.4 (-7.2, 4.3)	0.62219
N-acetylhistidine	Amino Acid	-17.9 (-36.5, 0.7)	0.05879
trans-urocanate	Amino Acid	-7.4 (-20.7, 5.9)	0.27298
2,3-dihydroxy-2-methylbutyrate	Amino Acid	15.5 (-1.7, 32.6)	0.07685
3-hydroxy-2-ethylpropionate	Amino Acid	-31.4 (-58, -4.7)	0.02111
3-hydroxyisobutyrate	Amino Acid	-28.2 (-45.3, -11.2)	0.00124
3-methyl-2-oxobutyrate	Amino Acid	-1.6 (-6.3, 3.2)	0.52423
3-methylglutaconate	Amino Acid	-39.1 (-60.2, -18)	0.00031
alpha-hydroxyisocaproate	Amino Acid	-8.8 (-31.8, 14.3)	0.45462
isoleucine	Amino Acid	51.8 (8.5, 95)	0.01906
isovalerylcarnitine (C5)	Amino Acid	8.2 (-9.7, 26.1)	0.37012
isovalerylglycine	Amino Acid	4.3 (-10.5, 19.1)	0.56490
5-(galactosylhydroxy)-L-lysine	Amino Acid	5.5 (-16.9, 28)	0.62821
5-hydroxylysine	Amino Acid	2.5 (-14.8, 19.8)	0.77356
glutarylcarnitine (C5-DC)	Amino Acid	-1.4 (-19.6, 16.8)	0.87923
N,N,N-trimethyl-5-aminovalerate	Amino Acid	7.5 (-14.7, 29.7)	0.50567
N6-acetyllysine	Amino Acid	-38.6 (-80.3, 3)	0.06900
cysteine s-sulfate	Amino Acid	-12.1 (-24.6, 0.3)	0.05647
methionine sulfone	Amino Acid	-7.8 (-28.1, 12.6)	0.45224
taurine	Amino Acid	-1 (-18.3, 16.2)	0.90528
(N(1) + N(8))-acetylspermidine	Amino Acid	-42.2 (-64.8, -19.6)	0.00028
4-acetamidobutanoate	Amino Acid	-16.7 (-36.8, 3.5)	0.10512

indoleacetate	Amino Acid	-0.8 (-22, 20.4)	0.94115
indolepropionate	Amino Acid	2.2 (-6.5, 10.9)	0.62073
tryptophan betaine	Amino Acid	-8.8 (-14.8, -2.8)	0.00441
3-methoxytyrosine	Amino Acid	-42.8 (-69.4, -16.2)	0.00169
N-acetyltyrosine	Amino Acid	10.8 (-8, 29.6)	0.25857
p-cresol glucuronide*	Amino Acid	-3.4 (-10.5, 3.6)	0.34078
phenol sulfate	Amino Acid	8.2 (-6.3, 22.8)	0.26738
thyroxine	Amino Acid	-33.7 (-64.9, -2.5)	0.03425
tyramine O-sulfate	Amino Acid	3.7 (-3.2, 10.5)	0.29463
argininate*	Amino Acid	11.1 (-2.1, 24.3)	0.10016
N-acetylarginine	Amino Acid	4 (-15.9, 23.9)	0.69309
N-methylproline	Amino Acid	-2.2 (-9.2, 4.7)	0.52751
N-acetylglucosamine/N-acetylgalactosamine	Carbohydrate	15.2 (-8.1, 38.5)	0.20066
mannitol/sorbitol	Carbohydrate	-4.9 (-16.6, 6.7)	0.40386
arabitol/xylitol	Carbohydrate	23.7 (-6.5, 54)	0.12383
arabonate/xylonate	Carbohydrate	2.8 (-18.1, 23.7)	0.79271
ribonate	Carbohydrate	0.2 (-21.7, 22.1)	0.98686
ribulonate/xylulonate*	Carbohydrate	-7.9 (-25.5, 9.7)	0.37812
gulonate*	Cofactors/Vitamins	7.7 (-15, 30.3)	0.50501
threonate	Cofactors/Vitamins	-11.2 (-29.4, 7)	0.22737
1-methylnicotinamide	Cofactors/Vitamins	-24.4 (-38.7, -10.1)	0.00088
quinolinate	Cofactors/Vitamins	2.4 (-13, 17.8)	0.75954
trigonelline (N'-methylnicotinate)	Cofactors/Vitamins	4.9 (-4, 13.7)	0.28104
pantothenate	Cofactors/Vitamins	-13 (-35.4, 9.4)	0.25341
beta-cryptoxanthin	Cofactors/Vitamins	-11.9 (-19.4, -4.5)	0.00178
carotene diol (2)	Cofactors/Vitamins	-10.2 (-23.6, 3.2)	0.13653
retinol (Vitamin A)	Cofactors/Vitamins	28.1 (2.6, 53.6)	0.03110
pyridoxate	Cofactors/Vitamins	-15.1 (-28.7, -1.4)	0.03058
deoxycarnitine	Lipid	-56 (-89.9, -22)	0.00131
ceramide (d18:1/14:0, d16:1/16:0)*	Lipid	9.5 (-7.2, 26.1)	0.26421
N-stearoyl-sphingadienine (d18:2/18:0)*	Lipid	32.4 (14.7, 50)	0.00034
palmitoyl-arachidonoyl-glycerol (16:0/20:4) [2]*	Lipid	55.8 (44.1, 67.4)	0.00000
palmitoyl-linoleoyl-glycerol (16:0/18:2) [1]*	Lipid	78.3 (66.3, 90.2)	0.00000
stearoyl-arachidonoyl-glycerol (18:0/20:4) [1]*	Lipid	39.8 (19.7, 59.9)	0.00011
N-oleoylserine	Lipid	-14.5 (-37.1, 8)	0.20583
adipoylcarnitine (C6-DC)	Lipid	-22.3 (-34.8, -9.8)	0.00051

laurylcarnitine (C12)	Lipid	-50.6 (-66.1, -35)	0.00000
linolenoylcarnitine (C18:3)*	Lipid	-14.7 (-30.3, 0.8)	0.06307
3-hydroxybutyroylglycine**	Lipid	-16.8 (-29.6, -4.1)	0.00989
N-palmitoylglycine	Lipid	-33.5 (-62.3, -4.7)	0.02266
hexadecanedioate (C16-DC)	Lipid	-40 (-56.7, -23.2)	0.00000
hexadecenedioate (C16:1-DC)*	Lipid	-30.9 (-46.3, -15.6)	0.00008
octadecadienedioate (C18:2-DC)*	Lipid	-15.5 (-26, -5)	0.00404
sebacate (C10-DC)	Lipid	-31.6 (-45.4, -17.8)	0.00001
tetradecanedioate (C14-DC)	Lipid	-35.9 (-51.2, -20.5)	0.00001
12,13-DiHOME	Lipid	-32.6 (-50, -15.1)	0.00028
2-hydroxylaurate	Lipid	-35.3 (-60.7, -9.8)	0.00670
2-hydroxynervonate*	Lipid	-44.1 (-69.1, -19)	0.00061
glycosyl ceramide (d18:1/20:0, d16:1/22:0)*	Lipid	-18.4 (-44.2, 7.5)	0.16329
glycosyl-N-stearoyl-sphingosine (d18:1/18:0)	Lipid	-11.4 (-39.7, 16.8)	0.42573
lactosyl-N-behenoyl-sphingosine (d18:1/22:0)*	Lipid	-39.6 (-55.4, -23.8)	0.00000
arachidate (20:0)	Lipid	-41.3 (-64, -18.7)	0.00037
margarate (17:0)	Lipid	-22.3 (-44.3, -0.4)	0.04645
1-linoleoyl-GPG (18:2)*	Lipid	38.7 (17.9, 59.5)	0.00029
1-arachidonylglycerol (20:4)	Lipid	23.4 (9.8, 37)	0.00081
1-linolenoylglycerol (18:3)	Lipid	46.6 (35.6, 57.6)	0.00000
1-oleoylglycerol (18:1)	Lipid	88.3 (73.9, 102.7)	0.00000
2-arachidonoylglycerol (20:4)	Lipid	16.8 (4.2, 29.5)	0.00907
1,2-dipalmitoyl-GPC (16:0/16:0)	Lipid	-38 (-80.2, 4.2)	0.07746
1-myristoyl-2-arachidonoyl-GPC (14:0/20:4)*	Lipid	47.8 (33.6, 62.1)	0.00000
1-stearoyl-2-oleoyl-GPC (18:0/18:1)	Lipid	76.1 (47.7, 104.5)	0.00000
1-palmitoyl-2-arachidonoyl-GPI (16:0/20:4)*	Lipid	71.6 (56.6, 86.6)	0.00000
1-(1-enyl-palmitoyl)-2-arachidonoyl-GPC (P-16:0/20:4)*	Lipid	-55.1 (-79.6, -30.5)	0.00001
1-(1-enyl-palmitoyl)-2-palmitoleoyl-GPC (P-16:0/16:1)*	Lipid	-69.3 (-92.8, -45.7)	0.00000
adrenate (22:4n6)	Lipid	-4.3 (-25.9, 17.3)	0.69417
glycochenodeoxycholate	Lipid	0.6 (-6.8, 8.1)	0.86952
taurocholate	Lipid	-3.1 (-10.5, 4.4)	0.41791
glycolithocholate sulfate*	Lipid	-8.9 (-17.1, -0.7)	0.03426
lithocholate sulfate (1)	Lipid	-5.8 (-13, 1.4)	0.11607
sphinganine-1-phosphate	Lipid	-6 (-17.8, 5.8)	0.31950
sphingomyelin (43:1)*	Lipid	-24.1 (-44.4, -3.8)	0.01989
sphingomyelin (d42:4)*	Lipid	-21.7 (-50.1, 6.7)	0.13457

7-alpha-hydroxy-3-oxo-4-cholestenoate (7-Hoca)	Lipid	-12.2 (-40.5, 16)	0.39506
allantoin	Nucleotide	18 (-2.4, 38.4)	0.08340
adenine	Nucleotide	14.1 (-7.5, 35.6)	0.20015
guanosine	Nucleotide	-1.1 (-6.9, 4.7)	0.70714
N2,N2-dimethylguanosine	Nucleotide	-17.3 (-57, 22.3)	0.39064
orotate	Nucleotide	-20.3 (-38.9, -1.7)	0.03290
orotidine	Nucleotide	9.7 (-13.7, 33.1)	0.41465
3-aminoisobutyrate	Nucleotide	-46.5 (-61.1, -31.8)	0.00000
5,6-dihydrothymine	Nucleotide	-41.8 (-70.1, -13.5)	0.00394
leucylalanine	Peptide	8.5 (0.3, 16.6)	0.04135
gamma-glutamylcitrulline*	Peptide	-9.4 (-21.8, 3)	0.13775
3-methoxycatechol sulfate (1)	Xenobiotics	3.4 (-3.8, 10.7)	0.35382
3-phenylpropionate (hydrocinnamate)	Xenobiotics	-4 (-11.9, 3.8)	0.31473
4-ethylphenylsulfate	Xenobiotics	-6.9 (-13.2, -0.6)	0.03297
4-hydroxyhippurate	Xenobiotics	3.2 (-9, 15.5)	0.60509
4-methylguaiacol sulfate	Xenobiotics	-4.7 (-13.3, 3.9)	0.28515
methyl-4-hydroxybenzoate sulfate	Xenobiotics	2.4 (-2.2, 7)	0.30293
2-naphthol sulfate	Xenobiotics	6.6 (-2.1, 15.3)	0.13589
3-hydroxypyridine sulfate	Xenobiotics	-1.6 (-10.9, 7.6)	0.72996
perfluorooctanoate (PFOA)*	Xenobiotics	-23.8 (-41.6, -6)	0.00873
sulfate*	Xenobiotics	-50.3 (-89.2, -11.4)	0.01133
hydroquinone sulfate	Xenobiotics	4.3 (-5.8, 14.4)	0.40026
2,3-dihydroxyisovalerate	Xenobiotics	8.6 (-0.7, 17.8)	0.06991
2-isopropylmalate	Xenobiotics	4.1 (-6.1, 14.3)	0.42778
2-piperidinone	Xenobiotics	2.2 (-5.1, 9.5)	0.55747
3,4-methyleneheptanoate	Xenobiotics	-22.8 (-33.9, -11.8)	0.00006
ergothioneine	Xenobiotics	-6 (-17.1, 5.2)	0.29326
erythritol	Xenobiotics	14 (-6.1, 34.1)	0.17062

[a] This table only shows metabolites that were identified. A total of 44 unknown metabolites are not shown.

[b] Estimates based on linear mixed effects models adjusted for participant age across visits, sex, and race/ethnicity (Hispanic, non-Hispanic white, non-Hispanic black, or non-Hispanic other). All models included a participant-specific random intercept.

[c] Displaying raw p-values from linear mixed effects models.

* Indicates tier 2 identification in which no commercially available authentic standard can be found; however, it was annotated based on accurate mass, spectral, and chromatographic similarity to tier 1 identified compounds.

Table S3. Sensitivity Analysis – Prospective associations between select metabolites associated with sugar sweetened beverage intake in childhood and triglycerides across childhood and adolescence *with additional adjustment for pubertal stage*.

Metabolite[a]	Pathway	Sub-Pathway	β (95% CI)[b]	P-value[c]
palmitoyl-linoleoyl-glycerol (16:0/18:2)*	Lipid	Diacylglycerol	77.0 (65.0, 89.0)	$<1.00 \times 10^{-7}$
hexadecenedioate (C16:1-DC)*	Lipid	Fatty Acid, Dicarboxylate	-31.6 (-46.8, -16.3)	5.66×10^{-5}
tetradecanedioate (C14-DC)	Lipid	Fatty Acid, Dicarboxylate	-35.4 (-50.7, -20.0)	7.72×10^{-6}
lactosyl-N-behenoyl-sphingosine (d18:1/22:0)*	Lipid	Lactosylceramides (LCER)	-38.2 (-54.0, -22.4)	2.79×10^{-6}
1-oleoylglycerol (18:1)	Lipid	Monoacylglycerol	87.2 (72.7, 101.7)	$<1.00 \times 10^{-7}$
1-linolenoylglycerol (18:3)	Lipid	Monoacylglycerol	45.9 (34.9, 57.0)	$<1.00 \times 10^{-7}$
1-stearoyl-2-oleoyl-GPC (18:0/18:1)	Lipid	Phosphatidylcholine (PC)	76.8 (48.5, 105.2)	1.63×10^{-7}
1-palmitoyl-2-arachidonoyl-GPI (16:0/20:4)*	Lipid	Phosphatidylinositol (PI)	70.7 (55.7, 85.8)	$<1.00 \times 10^{-7}$
1-(1-enyl-palmitoyl)-2-palmitoleoyl-GPC (P-16:0/16:1)*	Lipid	Plasmalogen	-69.8 (-93.3, -46.3)	$<1.00 \times 10^{-7}$

[a] This table only shows metabolites that were identified. A total of 2 unknown metabolites are not shown.

[b] Estimates based on linear mixed effects models adjusted for participant age across visits, sex, and race/ethnicity (Hispanic, non-Hispanic white, non-Hispanic black, non-Hispanic other), and pubertal stage across visits (pre-pubertal, pubertal, late-pubertal). All models included a participant-specific random intercept.

[c] Displaying raw p-values from linear mixed effects models. Only metabolites with a p-value less than the Bonferroni-corrected $p<0.000294$ (0.05/170 metabolite tests or 2.94×10^{-4}) and with an estimate in the same direction as with SSB intake in childhood are shown.

* Indicates tier 2 identification in which no commercially available authentic standard can be found; however, it was annotated based on accurate mass, spectral, and chromatographic similarity to tier 1 identified compounds.
